# Association of pelvic fracture patterns, pelvic binder use and arterial angio-embolization with transfusion requirements and mortality rates; a 7-year retrospective cohort study

**DOI:** 10.1186/s12893-017-0299-6

**Published:** 2017-11-09

**Authors:** Fabio Agri, Mylène Bourgeat, Fabio Becce, Kevin Moerenhout, Mathieu Pasquier, Olivier Borens, Bertrand Yersin, Nicolas Demartines, Tobias Zingg

**Affiliations:** 1 Department of Visceral Surgery, Lausanne University Hospital (Centre Hospitalier Universitaire Vaudois – CHUV), Rue du Bugnon 46, 1011 Lausanne, Switzerland; 2 Department of Emergency Medicine, Lausanne University Hospital (Centre Hospitalier Universitaire Vaudois - CHUV), Rue du Bugnon 46, 1011 Lausanne, Switzerland; 3 Department of Diagnostic and Interventional Radiology, Lausanne University Hospital (Centre Hospitalier Universitaire Vaudois - CHUV), Rue du Bugnon 46, 1011 Lausanne, Switzerland; 4 Department of Orthopedic Surgery, Lausanne University Hospital (Centre Hospitalier Universitaire Vaudois - CHUV), Rue du Bugnon 46, 1011 Lausanne, Switzerland

**Keywords:** Pelvic fracture classification, Circumferential compression device, Arterial angio-embolization, Packed red blood cell transfusion, Mortality

## Abstract

**Background:**

Pelvic fractures are severe injuries with frequently associated multi-system trauma and a high mortality rate. The value of the pelvic fracture pattern for predicting transfusion requirements and mortality is not entirely clear. To address hemorrhage from pelvic injuries, the early application of pelvic binders is now recommended and arterial angio-embolization is widely used for controlling arterial bleeding. Our aim was to assess the association of the pelvic fracture pattern according to the Tile classification system with transfusion requirements and mortality rates, and to evaluate the correlation between the use of pelvic binders and arterial angio-embolization and the mortality of patients with pelvic fractures.

**Methods:**

Single-center retrospective cohort study including all consecutive patients with a pelvic fracture from January 2008 to June 2015. All radiological fracture patterns were independently reviewed and grouped according to the Tile classification system. Data on patient demographics, use of pelvic binders and arterial angio-embolization, transfusion requirements and mortality were extracted from the institutional trauma registry and analyzed.

**Results:**

The present study included 228 patients. Median patient age was 43.5 years and 68.9% were male. The two independent observers identified 105 Tile C (46.1%), 71 Tile B (31.1%) and 52 Tile A (22.8%) fractures, with substantial to almost perfect interobserver agreement (Kappa 0.70-0.83). Tile C fractures were associated with a higher mortality rate (*p* = 0.001) and higher transfusion requirements (*p* < 0.0001) than Tile A or B fractures. Arterial angio-embolization for pelvic bleeding (*p* = 0.05) and prehospital pelvic binder placement (*p* = 0.5) were not associated with differences in mortality rates.

**Conclusions:**

Tile C pelvic fractures are associated with higher transfusion requirements and a higher mortality rate than Tile A or B fractures. No association between the use of pelvic binders or arterial angio-embolization and survival was observed in this cohort of patients with pelvic fractures.

## Background

Pelvic fractures are caused by high-energy forces and usually imply the presence of multiple injuries. Most occur in the setting of road traffic accidents (60%), falls (30%) and crush trauma (10%) [1]. Overall mortality from pelvic fractures associated with hemodynamic instability is around 30% [2, 3] and is caused by significant retroperitoneal bleeding or associated extra-pelvic injuries, most often of the chest or the central nervous system [4]. Potential pelvic bleeding sources may include bony fracture surfaces, the disrupted pelvic venous plexus and arterial bleeding from branches of the iliac vessels. Resuscitation, timely identification and adequate treatment of pelvic hemorrhage and significant associated injuries are essential [5].

The two most frequently used radiological classification systems for pelvic fractures are those proposed by Tile [6, 7] and by Young and Burgess [4, 8]. The Tile classification is based on the mode of mechanical pelvic ring instability. Type A fractures do not concern the pelvic ring per se and are stable, type B fractures are rotationally unstable whereas type C fractures are in addition vertically unstable. The Young-Burgess classification is based on the injury mechanism (lateral compression, anteroposterior compression and vertical shear forces). There is controversy about the clinical usefulness of both classification systems in terms of association of fracture patterns with the risk of significant bleeding and mortality, whether the Tile [9–13] or the Young-Burgess [3, 14–17] system is used. In the only study who compared the two, both classifications had similar predictive values for mortality, resuscitation fluid and transfusion requirements [18]. Published data show only low to moderate interobserver reliability of both systems [19–21]. Although readily available for unstable patients, plain radiography alone seems to be insufficient for evaluation of the posterior elements of the pelvic ring [8, 22, 23]. Computed tomography (CT) is the best imaging technique to detect injuries to the pelvic ring, bleeding and associated abdominal injuries [24, 25], but should be reserved for hemodynamically stable patients.

Interventions for hemorrhage control are application of pelvic binders [26], surgical stabilization with external fixation devices [27], arterial angio-embolization (AAE) [28, 29], extraperitoneal pelvic packing (EPP) [2, 30], and retrograde endovascular balloon occlusion of the aorta [31]. The efficiency of these measures in terms of mortality reduction and/or blood transfusion requirements is still unclear [32, 33]. Multiple management algorithms have been proposed, but treatment strategies depend on the local availability of human and technical resources such as interventional radiology (IR) [27, 34, 35].

Our primary objective was to assess a 7-year retrospective cohort of consecutive pelvic fracture patients with respect to the association of the pelvic fracture pattern according to the Tile classification with transfusion requirements and mortality. The secondary objective was to describe the correlation of pelvic binder application and AAE with patient outcome.

## Methods

This study was based on the prospective trauma registry of Lausanne University Hospital (Centre Hospitalier Universitaire Vaudois - CHUV), Switzerland, which includes all patients over 16 years of age admitted to the trauma resuscitation area of the emergency department (ED). All patients with a final diagnosis of pelvic fractures from January 2008 to June 2015 were included. Patients with isolated acetabular fractures were excluded. The study protocol was approved by the local institutional review board (Protocol No 2016-00927) and the manuscript prepared to conform to the Strengthening the Reporting of Observational Studies in Epidemiology (STROBE) guidelines [36].

Demographic data (age, gender), cause (accident, self-harm) and mechanism of injury (height of fall, type of road traffic accident, crush), placement of a pelvic binder, admission Glasgow Coma Scale (GCS) score, heart rate (HR), systolic blood pressure (SBP), lactate level, standard base deficit (SBD), Injury Severity Scale (ISS) score, Abbreviated Injury Scale (AIS) score by body region, intensive care unit (ICU) length of stay (LOS), mortality at 48 h and 30 days, units of packed red blood cells (PRBC) transfused, Tile classification of pelvic fracture, concomitant injuries, type of and time interval from arrival to diagnostic and therapeutic interventions were obtained. Shock index (SI) was calculated from HR/SBP. The primary outcomes were defined as total PRBC transfusion requirements and mortality.

For all emergency medical service (EMS) agencies of Western Switzerland, pelvic binder placement has been recommended in the prehospital setting since 2006 for patients with clinically suspected pelvic fractures after a high-energy trauma and for patients who are hemodynamically unstable without an obvious etiology. In the ED, binders were applied to patients who fulfilled the same criteria but arrived without a binder in place or in patients with a radiologically documented unstable pelvic fracture.

The Lausanne University Hospital trauma protocol follows the Advanced Trauma Life Support (ATLS®) [37] guidelines, specifically adapted to the local infrastructure and resources. Imaging in the trauma resuscitation bay includes plain films of the chest and pelvis. All hemodynamically unstable patients also undergo a Focused Abdominal Ultrasound for Trauma (FAST) exam. Stable patients next undergo whole-body contrast-enhanced CT followed by pelvic stabilization according to the attending orthopedic trauma surgeon’s decision. Unstable patients with a positive FAST are taken to the operating room (OR) for laparotomy with external fixation of the pelvis if indicated. In these patients, whole-body CT is performed after life-saving procedures and hemodynamic stabilization. AAE for pelvic injuries is either performed for initially stable or secondarily stabilized patients with arterial contrast extravasation seen on CT or for hemodynamically unstable patients with a documented pelvic fracture and no other source of instability (negative FAST, negative plain radiography of the chest and no external bleeding or neurogenic shock). When IR is unavailable for the latter patient group, EPP is performed in the OR. PRBC transfusion was initiated for patients with an estimated loss of >30% of the circulating blood volume.

Data were extracted from the prospective trauma registry of Lausanne University Hospital. When unavailable, data were collected from the electronic patient record. All available clinical, laboratory and imaging results were obtained and recorded during the initial phase of care in the ED. Patients underwent a plain radiograph of the pelvis (anterior-posterior view) in the trauma bay and/or a whole-body CT scan. Our standardized polytrauma CT protocol was performed using a 64-detector row CT unit (LightSpeed VCT; GE Healthcare, Milwaukee, WI, USA). To minimize analytical bias, all CT scans, and when unavailable pelvic x-rays were independently reviewed by a senior musculoskeletal radiologist (FB) and a senior orthopedic surgeon (KM) for categorization into the main (A-B-C), first (A/B/C *1-3*) and second-order (A/B/C 1/2/3*.1-3*) subgroups according to the Tile classification of pelvic fractures [6, 38]. All cases were matched and in case of discrepancy, a consensus agreement was reached with the chief orthopedic trauma surgeon (OB). For findings concerning patients undergoing surgery and/or IR for pelvic or abdominal injuries, operative and IR reports were consulted.

All data were recorded in an Excel spreadsheet (Microsoft Corp., Washington, DC, USA). Kappa values were calculated to assess interobserver reliability of the Tile classification [39]. Statistical analyses and graphics were performed using R software version 3.3.1 (R Foundation for Statistical Computing, Vienna, Austria) [40]. For qualitative variables, results are expressed in frequencies and percentages. For continuous variables, a measure of dispersion was given using median, with lower and upper interquartile ranges (IQR) or mean, with range and standard deviation (SD). When appropriate for better display, median followed by mean values in square brackets were given. Qualitative variables were compared using Fisher’s exact or χ^2^ test. Continuous variables were compared using Student’s *t*-test when distribution was bell shaped and using a Kruskal-Wallis or Mann-Whitney U-test if distribution was skewed. Differences were considered statistically significant for *p*-values <0.05. Variables from the univariate analysis differing at *p* < 0.2 were entered into a stepwise logistic regression model to identify independent risk factors for mortality.

## Results

During the study period, 240 patients with pelvic fractures were identified through the trauma registry. Twelve patients with isolated acetabular fractures were excluded, leaving 228 for analysis. For 193 patients (85%) CT images were available, whereas for 35 only conventional radiographs (anterior-posterior view) were obtained. The two observers (FB, KM) independently classified 158 (69%) of the fracture patterns identically into 22 of the 26 existing second-order subcategories of the Tile classification (Table 1).

**Table 1:** Tile classification and interobserver reliability for pelvic fractures (*n* = 228)

Categories and sub-categories			Spontaneous agreement n (%) 158 (69)	Classified by consensus n (%) 70 (31)	*p*
Main	1st order	2nd order			
*(k = 0.83)*	*(k = 0.75)*	*(k = 0.70)*			
A (*n* = 52)			46 (89)	6 (11)	0.001
	A1 (*n* = 2)		2 (100)	0	0.47
		A1.1 (*n* = 1)	1 (100)	0	1
		A1.2 (*n* = 1)	1 (100)	0	1
	A2 (*n* = 41)		36 (88)	5 (12)	0.002
		A2.1 (*n* = 18)	15 (83)	3 (17)	0.13
		A2.2 (*n* = 17)	15 (88)	2 (12)	0.06
		A2.3 (*n* = 6)	6 (100)	0	0.11
	A3 (*n* = 9)		8 (89)	1 (11)	0.28
		A3.1 (*n* = 1)	1 (100)	0	1
		A3.2 (*n* = 5)	4 (80)	1 (20)	0.51
		A3.3 (*n* = 3)	3 (100)	0	0.55
B (*n* = 71)			44 (62)	27 (38)	0.12
	B1 (*n* = 5)		2 (40)	3 (60)	0.33
		B1.1 (*n* = 3)	2 (67)	1 (33)	1
		B1.2 (*n* = 2)	0	2 (100)	0.09
	B2 (*n* = 56)		41 (73)	15 (27)	0.51
		B2.1 (*n* = 45)	33 (73)	12 (27)	0.59
		B2.2 (*n* = 11)	8 (73)	3 (27)	1
	B3 (*n* = 8)		1 (13)	7 (87)	0.001
		B3.1 (*n* = 1)	0	1 (100)	0.31
		B3.3 (*n* = 7)	1 (14)	6 (86)	0.004
	Bx^a^ (*n* = 2)		0	2 (100)	0.09
C (*n* = 105)			68 (65)	37 (35)	0.2
	C1 (*n* = 65)		45 (69)	20 (31)	1
		C1.1 (*n* = 1)	1 (100)	0	1
		C1.2 (*n* = 22)	13 (59)	9 (41)	0.33
		C1.3 (*n* = 42)	31 (74)	11 (26)	0.58
	C2 (*n* = 14)		7 (50)	7 (50)	0.13
		C2.2 (*n* = 8)	4 (50)	4 (50)	0.25
		C2.3 (*n* = 6)	3 (50)	3 (50)	0.37
	C3 (*n* = 25)		16 (64)	9 (36)	0.65
		C3.1 (*n* = 5)	2 (40)	3 (60)	0.33
		C3.2 (*n* = 2)	2 (100)	0	0.57
		C3.3 (*n* = 18)	12 (67)	6 (33)	0.49
	Cx^a^ (*n* = 1)		0	1 (100)	0.3

Fisher’s exact test was used. Unless stated otherwise, data are displayed as numbers (%). *k* = Cohen’s Kappa

^a^fracture type not further specified

No difference in the overall rate of agreement was found between conventional radiographs (66%) and CT images (70%) (*p* = 0.7). Of the 70 cases with disagreement, 32 concerned the main (*A-B-C*) categories (Kappa = 0.83), 20 the first-order (A/B/C *1-3*) subcategories (Kappa = 0.75) and 18 the second-order (A/B/C 1/2/3*.1-3*) subcategories (Kappa = 0.7). In the 70 discordant cases, a consensus agreement was found by reviewing the images with the chief orthopedic trauma surgeon (OB). In three cases, the fracture type could not be specified any further than into one of the main categories (one type C and two type B). As a result, 52 (23%) Tile A, 71 (31%) Tile B and 105 (46%) Tile C fractures were identified.

The demographics and characteristics of the study population, overall and by Tile fracture pattern are summarized in Table 2.

**Table 2:** Demographics and characteristics of the study population (*n* = 228), overall and by Tile fracture type

n (%)	All 228 (100)	Tile A 52 (23)	Tile B 71 (31)	Tile C 105 (46)	*p*
Age (years), median (IQR)	44 (26-58)	46 (25-59)	40 (28-57)	44 (27-58)	0.8
Male gender, n (%)	157 (69)	42 (81)	50 (70)	65 (62)	0.05
ISS, median (IQR)	22 (13-34)	17 (12-26)	17 (13-27)	29 (18-38)	<0.001
AIS head, median (IQR)	0 (0-2)	0 (0-3)	0 (0-2)	0 (0-2)	0.55
AIS chest, median (IQR)	2 (0-3)	1 (0-3)	2 (0-3)	2 (0-3)	0.12
AIS abdomen, median (IQR)	0 (0-2)	0 (0-2)	0 (0-2)	0 (0-2)	0.22
AIS spine, median (IQR)	0 (0-2)	0 (0-2)	0 (0-2)	2 (0-2)	0.01
GCS, median (IQR)	15 (9-15)	15 (5-15)	15 (12-15)	14 (9-15)	0.47
Admission SI (HR/SBP) > 1, n (%)	43 (19)	6 (12)	9 (13)	28 (27)	0.02
Admission SBP < 90 mmHg, n (%)	23 (10)	2 (3.8)	2 (2.8)	19 (18)	0.001
Base deficit (mEq/l), median (IQR)	4.2 (2.3-7.5)	3.1 (1.7-6.4)	3.8 (1.6-5.6)	5.6 (3.4-9.1)	0.001
Lactate (mmol/l), median (IQR)	2.4 (1.4-4)	1.9 (1.3-2.9)	2.2 (1.2-3.4)	2.9 (1.7-5.6)	0.001
Prehospital pelvic binder placed, n (%)	115 (50)	24 (46)	39 (55)	52 (50)	0.61
Arterial angio-embolization, n (%)	27 (12)	3 (5.8)	4 (5.6)	20 (19)	0.01
Total PRBC (units), median (IQR)	0 (0-3)	0 (0-0)	0 (0-0)	1 (0-7)	<0.001
ICU LOS (days), median (IQR)	0 (0-3)	0 (0-5)	0 (0-2)	1 (0-3)	0.37
48-h mortality, n (%)	30 (13)	2 (3.8)	5 (7)	23 (22)	0.001
30-day mortality, n (%)	39 (17)	7 (14)	7 (10)	25 (24)	0.04
*Injury mechanism:*					
Falls, n (%)	103 (45)	19 (37)	27 (38)	57 (54)	0.04
< 1 m	7 (3.1)	3 (5.8)	2 (2.8)	2 (1.9)	0.48
1-5 m	27 (12)	7 (14)	9 (13)	11 (11)	0.78
> 5 m	69 (30)	9 (17)	16 (23)	44 (42)	0.002
Road Traffic Accidents, n (%)	115 (50)	33 (64)	40 (56)	42 (40)	0.01
Cyclist	10 (4.4)	3 (5.8)	2 (2.8)	5 (4.8)	0.77
Motorcycle	32 (14)	12 (23)	10 (14)	10 (9.5)	0.08
Car	45 (20)	17 (33)	14 (20)	14 (13)	0.02
Pedestrian hit by vehicle	28 (12)	1 (1.9)	14 (20)	13 (12)	0.01
Crush, n (%)	10 (4.4)	0	4 (5.6)	6 (5.7)	0.21

*HR* Heart Rate, *ICU* Intensive Care Unit, *IQR* Interquartile Range, *LOS* Length of Stay, *PRBC* Packed Red Blood Cells, *SD* Standard Deviation, *SBP* Systolic Blood Pressure, *SI* Shock Index

Fisher’s exact test was used for categorical data, Kruskal-Wallis test for continuous data

Median patient age was 44 years (IQR 26-58) and 69% (*n* = 157) were male. Injury mechanisms were road traffic accidents (50%), falls from a height (45%) and crush trauma (4.4%). Accidents accounted for 84% of injuries, with the remainder being caused by intentional falls from a height (16%). Associated major injuries (AIS ≥3) most frequently concerned the chest (40%), the head (21%), the abdomen (18%) and the spine (10%). The median PRBC transfusion rate was 0 units (IQR 0-3) [mean, 2.5 units; range, 0-29; SD, 5] and the median ICU LOS was 0 days (IQR 0-3) [mean, 3.7 days; range, 0-56; SD, 7.8]. The overall mortality rate was 13% at 48 h (*n* = 30) and 17% at 30 days (*n* = 39).

There were no differences in patient age, gender or GCS among the three main fracture categories. Tile C fractures were associated with falls (*p* = 0.04), especially when from higher than five meters (*p* = 0.002), whereas Tile A and B fractures were more frequently observed after road traffic accidents (*p* = 0.01). Of these, Tile B fractures were more common in pedestrians hit by a vehicle (*p* = 0.01), whereas occupants of cars more often exhibited Tile A fractures (*p* = 0.02). Associated spine injuries were more frequent in patients with Tile C fractures (*p* = 0.01). Patients with Tile C fractures had significantly higher ISS (*p* < 0.0001), lactate levels (*p* = 0.001), SBD (*p* = 0.001) and SI (*p* = 0.02), and more frequently exhibited SBPs <90 mmHg (*p* = 0.001) than patients with Tile A and B fracture patterns.

**Fig. 1:**
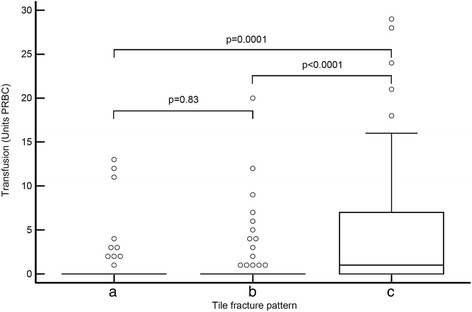
Boxplot diagrams showing median PRBC transfusion requirements for patients with Tile **a** (0 units; IQR, 0-0), **b** (0 units; IQR, 0-0) and **c** (1 unit; IQR, 0-7; *p* < 0.0001) fractures. PRBC = Packed Red Blood Cells

Patients with Tile C fractures had significantly higher median PRBC transfusion requirements (1 unit, IQR 0-7) [mean, 4.1 units; range, 0-29; SD, 6.2] than patients with type A (0 units, IQR 0-0) [mean, 1 unit; range, 0-13; SD, 2.9] or B fractures (0 units, IQR 0-0) [mean, 1.1 units; range, 0-20; SD, 3.2] (*p* < 0.0001) (Fig. 1).

Mortality rates were higher for Tile C fractures at 48 h (*p* = 0.0003) and 30 days (*p* = 0.01). No difference in mortality at 48 h was found between type A and B fractures (*p* = 0.7). Type B2 fractures were associated with increased survival at 48 h (*p* = 0.02) and 30 days (*p* = 0.02). Type A fractures were associated with increased survival at 48 h (*p* = 0.03), but not at 30 days (*p* = 0.5). Table 3 summarizes the mortality rates with univariate analysis for each pelvic fracture type according to the Tile classification.

**Table 3:** Association of 48-h mortality with pelvic fracture types

Tile Classification			Non-survivors *n* = 30 (13.2%)	Survivors *n* = 198 (86.8%)	*p*
A (*n* = 52)			2 (4)	50 (96)	0.03
	A1 (*n* = 2)		0	2	1
		A1.1 (*n* = 1)	0	1	1
		A1.2 (*n* = 1)	0	1	1
	A2 (*n* = 41)		2	39	0.12
		A2.1 (*n* = 18)	0	18	0.14
		A2.2 (*n* = 17)	2	15	1
		A2.3 (*n* = 6)	0	6	0.6
	A3 (*n* = 9)		0	9	0.37
		A3.1 (*n* = 1)	0	1	1
		A3.2 (*n* = 5)	0	5	0.62
		A3.3 (*n* = 3)	0	3	1
B (*n* = 71)			5 (7)	66 (93)	0.09
	B1 (*n* = 5)		0	5	0.62
		B1.1 (*n* = 3)	0	3	1
		B1.2 (*n* = 2)	0	2	1
	B2 (*n* = 56)		2 (4)	54 (96)	0.02
		B2.1 (*n* = 45)	1 (2)	44 (98)	0.02
		B2.2 (*n* = 11)	1	10	1
	B3 (*n* = 8)		1	7	1
		B3.1 (*n* = 1)	0	1	1
		B3.3 (*n* = 7)	1	1	0.25
	Bx^a^ (*n* = 2)		2	0	n.a.
C (*n* = 105)			23 (22)	82 (78)	0.0003
	C1 (*n* = 65)		12	53	0.19
		C1.1 (*n* = 1)	0	1	1
		C1.2 (*n* = 22)	4	18	0.5
		C1.3 (*n* = 42)	8	34	0.31
	C2 (*n* = 14)		4	10	0.09
		C2.2 (*n* = 8)	1	7	1
		C2.3 (*n* = 6)	3 (50)	3 (50)	0.03
	C3 (*n* = 25)		6	19	0.11
		C3.1 (*n* = 5)	1	4	1
		C3.2 (*n* = 2)	0	2	1
		C3.3 (*n* = 18)	5	13	0.07
	Cx^a^ (*n* = 1)		1	0	n.a.

Fisher’s exact test was used. Categorical data are displayed as numbers and (%) for main categories and significant subcategories

^a^fracture type not further specified

Pelvic binders had been applied in the field to 115 patients (50%) and to 29 more (13%) upon arrival in the ED. They were placed with comparable frequency among the three main fracture types (*p* = 0.61). Placement of pelvic binders was not associated with differences in admission SBP, HR, SI, lactate level, SBD, transfusion requirements, need for AAE or mortality rates at 48 h or 30 days, compared to the absence of pelvic binders, even when selecting unstable fracture types (B1, B3 and C) only (Table 4).

**Table 4:** Patient characteristics with and without pelvic binders (Tile B1, B3 and C fractures)

n (%)	Binder 61 (52)	No binder 57 (48)	*p*
ISS, median (IQR)	26 (17-38)	29 (18-38)	0.99
Admission SI (HR/SBP) > 1, n (%)	16 (26)	14 (25)	1
Admission SBP < 90 mmHg, n (%)	9 (15)	11 (19)	0.63
Base deficit (mEq/l), median (IQR)	4.5 (2.8-7.9)	5.8 (3.5-9.1)	0.29
Lactate (mmol/l), median (IQR)	2.7 (1.5-3.4)	2.9 (1.8-4.9)	0.81
Arterial angio-embolization (for pelvis), n (%)	5 (8)	7 (12)	0.55
Total PRBC (units), median (IQR)	0 (0-6)	2 (0-6)	0.91
48-h mortality, n (%)	14 (23)	10 (18)	0.5
30-day mortality, n (%)	15 (25)	11 (19)	0.51

*HR* Heart Rate, *PRBC* Packed Red Blood Cells, *SBP* Systolic Blood Pressure, *SI* Shock Index (HR/SBP)

Fisher’s exact test was used for categorical data, Mann-Whitney U test for continuous non-parametric variables

The median time interval from ED arrival to CT scan was 28 min (IQR 21-36) (*n* = 193), without any difference between survivors (28 min, IQR 20-35) and non-survivors (29.5 min, IQR 23-42) (*p* = 0.15). After initial workup and resuscitation in the trauma bay, 74 patients (33%) were taken to the OR, 17 (8%) to the IR suite and 28 (12%) to the ICU. Stable patients not requiring immediate intervention (*n* = 106, 46%) underwent a complete workup in the ED before transfer to the ward. The remaining three patients (1.3%) died before reaching any of the aforementioned destinations.

Of the 74 patients initially taken to the OR, 25 (34%) underwent temporary external fixation of the pelvis. Combined laparotomy for intra-abdominal injuries was performed in three of these, but in none an EPP was done. In eight patients external fixation was followed by AAE. Laparotomy without surgical stabilization of the pelvis was the first procedure in eight patients (11%), with one of these undergoing EPP (with a pelvic binder in place). The 41 remaining patients (55%) underwent urgent neurosurgical, thoracic or non-pelvic orthopedic procedures.

A total of 27 (12%) patients underwent AAE, of which 17 (63%) as their initial treatment. Significantly more patients with Tile C fractures underwent AAE for bleeding control (*p* = 0.01). Patients who underwent AAE for a pelvic bleeding source (*n* = 22) had a median ISS of 38 (IQR 29-43) and their median time interval from ED arrival to AAE was 105 min (IQR 79-124). Of these, seven had associated non-pelvic abdominal or thoracic bleeding sources. Median time interval to AAE was 98 min (IQR 74-120) in survivors and 108 (IQR 94-129) in non-survivors at 48 h (*p* = 0.20). The difference in median time interval to AAE was highest between survivors (80 min) and non-survivors (105 min) with a SBP <90 mmHg (*p* = 0.21). Five patients underwent AAE for isolated non-pelvic bleeding. Patients who underwent arterial AAE for pelvic bleeding had higher median PRBC transfusion requirements (6.5 units; IQR, 2-12) [mean, 8.1 units; range 0-28; SD, 7.4] than patients without AAE (0 units; IQR 0-2) [mean, 1.8 units; range, 0-29; SD, 4.3] (*p* < 0.0001).

The demographics and characteristics of survivors and non-survivors at 48 h are summarized in Table 5.

**Table 5:** 48-h mortality, characteristics of non-survivors (*n* = 30) and survivors (*n* = 198)

n (%)	Non-survivors 30 (13)	Survivors 198 (87)	*p*
Age (years), median (IQR)	62 (30-78)	41 (25-55)	0.001
Male gender, n (%)	16 (53)	141 (71)	0.06
ISS, median (IQR)	38 (26-49)	21 (13-29)	<.0001
AIS head, median (IQR)	3 (0-5)	0 (0-2)	<.0001
AIS chest, median (IQR)	3 (2-4)	2 (0-3)	0.001
AIS abdomen, median (IQR)	1 (0-3)	0 (0-2)	0.06
AIS spine, median (IQR)	2 (0-2)	0 (0-2)	0.37
GCS, median (IQR)	3 (3-12)	15 (12-15)	<.0001
Admission SI (HR/SBP) > 1, n (%)	11 (37)	32 (16)	0.004
Admission SBP < 90 mmHg, n (%)	8 (27)	15 (7.6)	0.004
Base deficit (mEq/l), median (IQR)	9 (3.9-17)	4 (1.9-6.8)	0.0004
Lactate (mmol/l), median (IQR)	4.5 (2.5-9.3)	2.2 (1.4-3.7)	0.0001
Prehospital pelvic binder placed, n (%)	19 (63)	96 (49)	0.17
Time to CT (minutes), median (IQR)	29.5 (23-42)	28 (20-35)	0.15
Angio-embolization, n (%)	7 (23)	20 (10)	0.06
Angio-embolization (for pelvis), n (%)	6 (20)	16 (8)	0.05
Time to embolization, all (minutes), median (IQR)	105 (90-135)	110 (76-169)	0.47
Time to embolization, for pelvis (minutes), median (IQR)	108 (94-129)	98 (74-120)	0.2
Time to embolization, SBP < 90 mmHg (minutes), median (IQR)	105 (98-116)	80 (70-95)	0.21
Total PRBC (units), median (IQR)	7 (2-12)	0 (0-1)	<.0001
*Tile fracture type:*			
Tile A, n (%)	2 (3.8)	50 (96)	0.03
Tile B, n (%)	5 (7)	66 (93)	0.09
Tile C, n (%)	23 (22)	82 (78)	0.0003
*Injury mechanism:*			
Falls, n (%)	17 (57)	86 (43)	0.12
< 1 m	0	7 (3.5)	0.6
1-5 m	2 (6.7)	25 (13)	0.4
> 5 m	15 (50)	54 (27)	0.02
Road Traffic Accidents, n (%)	13 (43)	102 (52)	0.3
Cyclist	2 (6.7)	8 (4)	0.62
Motorcycle	1 (3.3)	31 (16)	0.09
Car	5 (17)	40 (20)	0.81
Pedestrian hit by vehicle	5 (17)	23 (12)	0.55
Crush, n (%)	0	10 (5.1)	0.24

*CT* Computed Tomography, *HR* Heart Rate, *PRBC* Packed Red Blood Cells, *SBP* Systolic Blood Pressure, *SD* Standard Deviation, *SI* Shock Index

Fisher’s exact test was used for categorical data, Mann-Whitney test for continuous non-parametric variables

The median survival time was 7.6 h (IQR 2.9-47) for non-survivors, of which 30 (77%) died within 48 h from admission. Of these, 15 (50%) died from severe head injury and 15 (50%) from exsanguination which was due to pelvic injuries in seven (23%), chest injuries in four (13%), abdominal injuries in three (10%) and an extremity injury in one patient (3.3%). The remaining nine patients (23%) died between 48 h and 30 days after admission. Of these, eight died from severe head injury and one from ventilator-associated pneumonia. Application of a pelvic binder (in the field or in the ED) was not associated with decreased mortality at 48 h, even when patients with stable fracture types (A and B2) were excluded (*p* = 0.5). AAE was performed in 23% of non-survivors versus 10% of survivors (*p* = 0.06). The median ISS was 43 (IQR 34-50) in non-survivors versus 36 (IQR 29-41) in survivors who underwent AAE (*n* = 27, *p* = 0.04). Non-survivors had significantly higher median PRBC transfusion requirements (7 units, IQR 2-12) [mean, 7.1 units; range, 0-21; SD, 5.8] than survivors (0 units, IQR 0-1) [mean, 1.8 units; range, 0-29; SD, 4.4] (*p* < 0.0001).

**Fig. 2:**
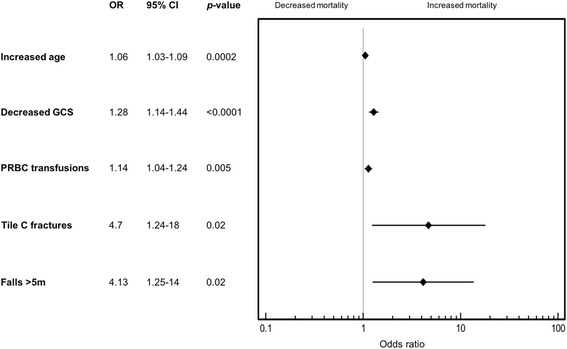
Forest plot showing factors associated with 48-h mortality after multivariate analysis. CI = Confidence interval, OR = Odds ratio, PRBC = Packed Red Blood Cells

After multivariate analysis, factors associated with 48-h mortality were increased patient age (odds ratio, 1.06; 95% CI, 1.03-1.09; *p* = 0.0002), decreased GCS (odds ratio, 1.28; 95% CI, 1.14-1.44; *p* < 0.0001), number of PRBC transfused (odds ratio, 1.14; 95% CI, 1.04-1.24; *p* = 0.005), falls from >5 m (odds ratio, 4.13; 95% CI, 1.25-14; *p* = 0.02) and Tile C fracture pattern (odds ratio, 4.7; 95% CI, 1.24-18, *p* = 0.02) (Fig. 2).

## Discussion

In the present series, patients with Tile C fractures had a significantly higher mortality and PRBC transfusion rate than patients with Tile A or B fractures. Differences in mortality rates and risk of bleeding related to the Tile fracture patterns have been described in the literature before, but not consistently. O’Sullivan et al. [10] analyzed 35 fatalities among a series of 174 patients with pelvic fractures but found no association with the Tile fracture type (*p* = 0.07). Lunsjo et al. [11] observed an association of mortality with ISS but not with the pelvic fracture pattern. Anandakumar et al. [12] observed a link between Tile fracture patterns and arterial contrast extravasation with need for AAE. Hussami et al. [13] found a significant correlation between the Tile fracture type and arterial, but not venous bleeding in a series of trauma patients who underwent postmortem CT. Rommens and Hessmann [9] not only found a higher mortality rate, but also worse functional outcomes when comparing type C with type B fractures. Recently, Costantini et al. [3] found Young-Burgess Type III anterior-posterior compression fractures to be at greatest risk for significant bleeding in a prospective multicenter study including patients who were in shock on admission.

Surprisingly, we only observed five type B1 fractures in contrast to 56 type B2 fractures in the present cohort. Bonner et al. [41] have shown that correctly placed pelvic binders could effectively reduce unstable pelvic ring injuries. This is supported by a recently published study by Swartz et al. [42], who have shown that pelvic binders could mask the true nature of the fracture pattern and in particular made Tile C and B3 fractures look like B2 fractures. Since in the present series about half of the fractures were classified based on imaging with pelvic binders in place, it can be hypothesized that some B1 fractures may have been classified as B2 fractures.

There was a significantly lower than average mortality rate in patients with type B2 fractures in the present series. Although not described in the literature before, this makes sense from a biomechanical point of view since there is less vascular disruption and the virtual pelvic space is reduced in analogy to the therapeutic principle of pelvic binders.

Similar as in the study by Lunsjo et al. [11], most deaths in the present series (77%) were caused by associated injuries, not the pelvic fracture itself (23%). Severe head injuries were responsible for the major part of mortality, as reflected by the median GCS of 3 and median AIS head of 3 in non-survivors. GCS was not associated with any of the Tile fracture patterns though. Death from hemorrhagic shock was most often caused by unstable pelvic fractures in the present series, as opposed to a study by Vaidya et al. [43] in which exsanguination was most often caused by bleeding from multiple areas and rarely from pelvic injury alone.

No association between the use of pelvic binders and decreased mortality was observed in the present study. Only 50% of patients with pelvic fractures arrived in the ED with a circumferential compression device in place. Toth et al. [26] have observed a similar rate in their study. Pelvic binders are safe to use and seem to provide efficient mechanical stabilization [44]. Their potential for limiting ongoing bleeding is less clear [45]. Only one study by Croce et al. [46] found decreased transfusion requirements and a positive effect on hemodynamics for patients with unstable pelvic fractures when binders were placed. In the present series, an association between the use of pelvic binders and transfusion requirements, vital signs, metabolic parameters (base deficit, lactate) or mortality rate could not be established.

Arterial bleeding has been reported in up to 15% of hemodynamically unstable pelvic fractures and AAE may reduce the need for PRBC transfusion [47]. In the present study, patients with Tile C fracture patterns were hemodynamically unstable, underwent AAE and required PRBC transfusions significantly more often than patients with other fracture types. A study by Hauschild et al. [48] found that AAE, compared to conventional measures of bleeding control, was not associated with a lower overall mortality rate in patients with pelvic fractures and associated vascular injuries, but death from exsanguination was significantly less frequent in the AAE group than in the conventional group. We observed no survival benefit or decreased transfusion requirements for patients who underwent AAE in the present cohort. There even was a slight decrease in survival. This is probably because patients who underwent AAE had a significantly higher overall trauma burden, as reflected by their ISS, than those who did not.

The time factor may play an important role in the outcome of AAE. Tanizaki et al. [33] have observed a reduction in mortality for hemodynamically unstable pelvic fracture patients if the door-to-needle time was less than 60 min. Hemodynamically unstable patients had a median time interval from ED arrival to AAE that was 10 min shorter for survivors than for non-survivors, but this difference was not statistically significant due to the small sample size. Osborn et al. [34] have compared AAE with EPP and have found significantly shorter time intervals between ED arrival and bleeding control for EPP. Time intervals from ED arrival to AAE found in the literature vary, with a median time interval to AAE of 280 min in one recent study from a high volume trauma center [49].

For any imaging-based classification system to be useful there should be a high level of interobserver agreement. When applied to our patient population, a substantial to almost perfect interobserver reliability of the Tile classification system was observed. This contrasts with previous studies where only low to moderate levels of agreement for the Tile classification system were found [19–21]. The high interobserver agreement in the present study may be related to the presence of only two observers, compared to six [19], five [20] and three [21] observers in the previous studies. As in the study by Koo et al. [19], the increase in interobserver reliability when using CT imaging was not significant in the present series. Interestingly, of the 70 cases with interobserver disagreement in the present series, most (*n* = 32) occurred already at the main (A/B/C) classification. As expected, consensual first and second order sub-classification was straightforward for type A fractures, but not for type B and C fractures. Consensus on first order sub-classification was worst for type C fractures, and on second order for type B fractures.

Only one patient in this cohort underwent EPP. According to the Lausanne University Hospital polytrauma management algorithm, hemodynamically unstable patients with a pelvic fracture, negative chest radiograph and no free abdominal fluid on FAST are taken to IR for AAE. The reason why this patient went to the OR for EPP was that the IR team was already occupied with AAE for another unstable pelvic fracture patient. In this algorithm, EPP is used for primary bleeding control only when IR is unavailable.

The study has several limitations. It reports a single center experience with relatively uniform practices based on local treatment protocols. Given its retrospective nature, information bias was inherently present. Also, data accuracy was subject to documentation errors in the trauma registry and patient record. There were only two observers for pelvic fracture classification. Since interobserver agreement was substantial to almost perfect, this number can be considered as adequate. Furthermore, assessment of interobserver reliability was not a goal of the present study. Finally, the fact that no significant association of pelvic binder or AAE use and mortality was observed in our cohort must be interpreted cautiously. Both interventions were performed for patients who had a higher pelvic and overall trauma burden, as reflected by the slightly higher observed mortality rates for these patients. Thus, the absence of any association of AAE and/or pelvic binder use with mortality does not allow to conclude on the absence of a treatment effect of those interventions, but most likely represents a selection bias.

## Conclusions

In conclusion, patients with Tile C pelvic fractures had significantly higher transfusion requirements than Tile A and B fractures, between which there was no difference. 48-h mortality rates were highest for Tile C fractures and lowest for Tile A and Tile B2 fractures. AAE was more frequently performed for Tile C fractures, but no association with patient survival or transfusion requirements were found. Use of circumferential pelvic compression devices was not associated with patient hemodynamics, physiological status, transfusion requirements or mortality in the present cohort.

## Abbreviations

(AAE): Arterial angio-embolization
(AIS): Abbreviated injury scale
(CI): Confidence interval
(CT): Computed tomography
(ED): Emergency department
(EPP): Extraperitoneal pelvic packing
(FAST): Focused abdominal ultrasound for trauma
(GCS): Glasgow coma scale
(HR): Heart rate
(ICU): Intensive care unit
(IQR): Interquartile range
(IR): Interventional radiology
(ISS): Injury severity scale
(LOS): Length of stay
(PRBC): Packed red blood cells
(SBD): Standard base deficit
(SBP): Systolic blood pressure
(SD): Standard deviation
(SI): Shock index
